# Kinetics of cellular immune response to SARS-CoV-2 vaccine boosters in dialysis and kidney transplant patients without infection

**DOI:** 10.1590/2175-8239-JBN-2025-0206en

**Published:** 2026-03-23

**Authors:** Roberto Matias Souza, Julia Soares Reis, Helio Tedesco-Silva, Lúcio Requião-Moura, José Medina-Pestana, Renato Demarchi Foresto

**Affiliations:** 1 Universidade Federal de São Paulo, Departamento de Medicina, Disciplina de Nefrologia, São Paulo, SP, Brazil.; 2 Hospital do Rim, Fundação Oswaldo Ramos, São Paulo, SP, Brazil.

**Keywords:** Immunity, Cellular, SARS-CoV-2, Kidney Transplantation, COVID-19 Vaccines, Dialysis

## Abstract

**Introduction:**

Dialysis patients and kidney transplant recipients (KTRs) present impaired immune response to COVID-19 vaccines. Although vaccination remains the primary strategy to reduce mortality, data on the cellular immune response kinetics in these populations are limited. This study evaluated the kinetics of vaccine-induced cellular immunity to SARS-CoV-2 in dialysis and KTRs without prior infection.

**Methods:**

This is a *post-hoc* analysis of a prospective, observational, single-center study comparing the cellular immunity kinetics induced by SARS-CoV-2 vaccination in adult patients undergoing kidney transplant (n = 77) and patients on dialysis (n = 92). Those who had COVID-19 were excluded. Blood samples were collected at screening and at 1, 3, 6, and 12 months to assess SARS-CoV-2-specific T-cell responses using a commercial Interferon-gamma release assay (IGRA, QuantiFERON SARS-CoV-2 RUO), with results classified as positive, negative, or indeterminate. Comparisons were performed using chi-square and Mann-Whitney tests.

**Results:**

Positive IGRA results were infrequent in transplant and dialysis patients over time (Screening: 8.1% vs. 23.2%; M1: 14.1% vs. 16.5%; M3: 10% vs. 7.1%; M6: 1.5% vs. 2.5%; M12: 15.2% vs. 13.4%), but indeterminate results were common (Screening: 82.4% vs. 17.8%; M1: 71.8% vs. 42.4%; M3: 62.9% vs. 47.1%; M6: 69.1% vs. 58.8%; M12: 30.3% vs. 19.4%), particularly among KTRs, although they received more vaccine boosters (95 vs. 63; p < 0.001). No correlation was observed between the number of vaccine doses and IGRA positivity.

**Conclusion:**

Despite multiple vaccine boosters, dialysis and kidney transplant patients exhibited limited and variable cellular immune responses, highlighting the need for improved vaccination and monitoring strategies in these high-risk populations.

## Introduction

The COVID-19 pandemic led to millions of deaths worldwide, with Brazil being one of the most affected countries. This event posed a significant risk to kidney transplant recipients (KTRs), who experienced a fatality rate of over 24%, compared to around 3% in the general population at the onset of the pandemic^1^.

Despite ongoing efforts to develop effective and safe vaccines, dialysis patients and KTRs continued to face high mortality rates even after vaccination^1,2^. Only after the widespread application of booster doses and the emergence of the Omicron variant did an improvement in disease outcomes in these populations occur^3^.

Following vaccination, KTRs exhibit a weaker cellular and humoral immune response than the general population and patients in other chronic kidney disease (CKD) stages^4^. This situation raised concerns about the safety of performing transplants during the pandemic and whether the high fatality rates, even among younger individuals with fewer comorbidities, were related to the immunosuppressive regimen to which they are subjected^1^.

Much focus has been placed on the kinetics of the humoral immune response acquired through SARS-CoV-2 vaccination in KTRs^5,6^. The humoral response is known to be weakened in KTRs due to the use of immunosuppressive drugs that reduce the mounting of an efficient immune response against the pathogen, such as the inhibition of lymphocyte activation and interaction with antigen-presenting cells, and decreased memory B cell responses^7^. Compared to patients with end-stage kidney disease remaining on dialysis, KTRs had lower SARS-CoV-2 IgG titers over time, even while receiving more boosters, and exhibited a higher incidence of COVID-19^5^. However, the cellular immune response after vaccination is an important aspect to consider in immune defense mechanisms against pathogens, which has been little explored in the context of SARS-CoV-2 infection. This response has already proven effective in both KTRs and healthy individuals, and it has conferred disease protection even in cases where the humoral component of the immune response was not prominent^8,9^.

Thus, this study aims to assess the impact of kidney transplant immunosuppression on the kinetics of the cellular immune response acquired through SARS-CoV-2 vaccination in patients who did not develop COVID-19. It was conducted exclusively in adult patients, and the findings may not be generalizable to pediatric populations.

## Methods

### Study Design and Population

**Figure 1:**
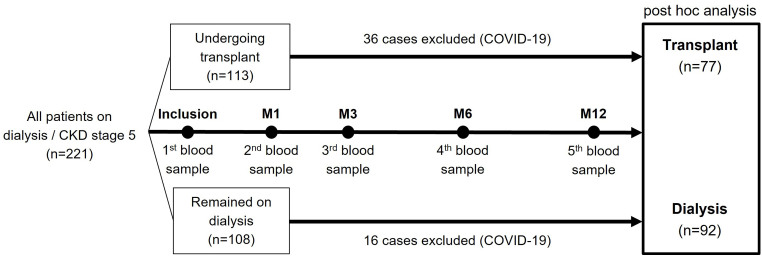
Flowchart of the study population.

This is a *post hoc* analysis of a prospective, observational, single-center study, previously published, comparing the kinetics of the immune response acquired through SARS-CoV-2 vaccination in patients undergoing kidney transplant and those remaining on dialysis^5^. The previously published cohort investigated the kinetics of the humoral immune response to SARS-CoV-2 vaccination in KTRs and dialysis patients^5^. In this current analysis, we aim to evaluate the effect of kidney transplant immunosuppression, focusing exclusively on the kinetics of the cellular immune response, assessed through IGRA, acquired through SARS-CoV-2 vaccination in patients who did not develop COVID-19. This design allowed us to isolate the vaccine-induced T-cell response without the confounding influence of natural infection (Figure 1).

The study enrolled adult patients over 18 years of age with end-stage CKD who were fully vaccinated, had seroconverted, and had no prior diagnosis of COVID-19. All participants were on the waiting list for kidney transplantation, and both cohorts were recruited concurrently throughout the study period. Patients who underwent transplantation were assigned to the transplant group, while those who remained on dialysis were allocated to the dialysis group (Figure 1). The transplant group included recipients of kidneys from both living and deceased donors. Exclusion criteria included patients who tested negative for SARS-CoV-2 IgG at enrollment, individuals living with HIV, and those receiving cancer treatment. The study period during the pandemic saw a predominance of the circulation of the Omicron variant of SARS-CoV-2 in a context of high vaccination coverage in the population. The Kidney Donor Profile Index is a score that summarizes the expected quality of a deceased donor kidney based on donor characteristics.

### Study Procedures

This study was approved by the Institutional Review Board (IRB) of the Federal University of São Paulo (approval number 5.156.043). All eligible participants provided written informed consent before enrollment. The study adhered to the principles of the Declaration of Helsinki and followed the Good Clinical Practice guidelines. Each patient was monitored for 12 months from the date of inclusion, with blood samples collected shortly after enrollment and subsequently at 1, 3, 6, and 12 months to assess the immune response to SARS-CoV-2 (Figure 1). For participants in the transplant group, the initial blood sample was obtained before initiating immunosuppressive therapy. The study did not modify the standard immunosuppressive regimens used in kidney transplantation. It also did not recommend vaccination or the timing of additional booster doses. All participants received SARS-CoV-2 vaccines through the national public health system under the Ministry of Health’s guidelines. All kidney transplants and follow-ups of dialysis patients included in the study were carried out at our center.

### Cellular Immunity Assessment

The cellular immune response was assessed by qualitative detection of IFN-gamma produced by CD4+ and CD8+ T cell responses to SARS-CoV-2 peptides using the QuantiFERON SARS-CoV-2 RUO Starter Set (QIAGEN). A positivity threshold was defined as ≥0.35 IU/mL. Blood samples were collected into three separate tubes: (1) Nil Tube, which contains only heparin and serves as a negative control; (2) Ag3 Tube, which includes SARS-CoV-2-specific antigens to stimulate an immune response; and (3) Mitogen Tube, which contains phytohemagglutinin as a positive control^10^. After collection, the tubes were gently mixed and incubated at 37 ± 1 °C for 16 hours. The samples were then centrifuged at 3,000 rpm for 15 minutes to separate the plasma, which was stored at –70 °C for later analysis. The quantification of IFN-γ levels in plasma was performed using an ELISA. The QuantiFERON SARS-CoV-2 ELISA utilizes a recombinant human IFN-γ standard that is calibrated against a reference preparation (NIH Ref: Gxg01-902-535). Test results were expressed in IU/mL, based on a standard curve generated from serial dilutions of the reference standard. The Mitogen Tube served as a positive control to confirm sample responsiveness, while the Nil Tube accounted for baseline IFN-γ production^10^.

### Vaccines Administered and the National Immunization Program

The Brazilian national COVID-19 vaccination campaign began on January 17, 2021, starting with the administration of CoronaVac, an inactivated virus vaccine^11^. The vaccination process prioritized high-risk groups to receive the vaccine first. In addition to CoronaVac, other vaccines became available and were distributed by the National Immunization Program: ChAdOx1 nCoV-19 (AstraZeneca), which employs a viral vector platform; BNT162b2 (Comirnaty, Pfizer-BioNTech), which uses messenger RNA technology; and Ad26.COV2.S (Janssen), another viral vector-based vaccine^12,13^.

### Institutional Protocol of Immunosuppression

The immunosuppressive protocol includes both induction and maintenance therapies. At the *Hospital do Rim*, all kidney transplant recipients receive 1,000 mg of methylprednisolone intraoperatively, followed by a single 3 mg/kg dose of anti-thymocyte globulin administered on the day after surgery. Maintenance immunosuppression is based on a combination of a corticosteroid, a calcineurin inhibitor, and a third agent, either azathioprine or mycophenolate sodium, chosen according to the recipient’s immunological risk profile and the type of donor.

### Statistical Analysis

Qualitative variables were summarized in frequency distributions, expressed as absolute numbers and percentages, and groups were compared using the chi-square or Fisher’s exact tests. The Kolmogorov-Smirnov normality test was performed to verify whether the probability distribution associated with the set of all quantitative variables could be approximated by the normal distribution. Quantitative variables were presented as median and interquartile range for nonparametric variables and as mean and standard deviation for parametric variables. Differences between groups were analyzed using the Mann-Whitney test.

All statistical analyses were performed using the Statistical Package for the Social Sciences (SPSS) version 29 (IBM Corp., Released 2022. IBM SPSS Statistics for Windows, Version 29.0, Armonk, NY: IBM Corp.). A p-value < 0.05 was considered statistically significant, with a 95% confidence interval.

## Results

### Demographics

A total of 221 patients were included in the original study, 113 in the transplant group (Tx) and 108 in the dialysis group. During the 12-month follow-up, 36 KTRs and 16 dialysis patients developed COVID-19 and were censored, resulting in 77 (Tx) and 92 (dialysis) patients included in these analyses (Figure 1).

The median recipient age (45.4 vs. 54.0 years; p < 0.001) and dialysis duration (29.1 vs. 50.2 months; p < 0.009) were higher in the dialysis group. The other demographic characteristics were similar between the groups and are summarized in Table 1. In both groups, most patients were male (54.4% vs. 58.7%; p = 0.588), white (59.7% vs. 57.6%; p = 0.763), had hypertension (83.1% vs. 87.0%; p = 0.484), diabetes (19.5% vs. 27.2%; p = 0.241), presented indeterminate CKD (40.3% vs. 28.3%; p = 0.111), and underwent hemodialysis as renal replacement therapy (80.5% vs. 85.9%; p = 0.085) (Table 1).

**Table 1:** Demographic characteristics

	Total N = 169	Transplant N = 77	Dialysis N = 92	p
**Age**, years (IQR)	50.4 (39.2–59.4)	45.4 (36.1–57.0)	54.0 (45.4–63.0)	<0.001
**Male sex**, N (%)	96 (56.8)	42 (54.5)	54 (58.7)	0.588
**Ethnicity**, N (%)				0.763
*White*	99 (58.6)	46 (59.7)	53 (57.6)	
*Mixed*	49 (29.0)	23 (29.9)	26 (28.3)	
*Black*	21 (12.4)	8 (10.4)	13 (14.1)	
**Hypertension**, N (%)	144 (85.2)	64 (83.1)	80 (87.0)	0.484
**Diabetes Mellitus**, N (%)	40 (23.7)	15 (19.5)	25 (27.2)	0.241
**CKD etiology**, N (%)				0.111
*Indeterminate*	57 (33.7)	31 (40.3)	26 (28.3)	
*Glomerulonephritis*	41 (24.3)	22 (28.6)	19 (20.7)	
*Diabetes Mellitus*	30 (17.8)	13 (16.9)	17 (18.5)	
*Hypertension*	17 (10.1)	4 (5.2)	13 (14.1)	
*Polycystic kidney disease*	15 (8.9)	4 (5.2)	11 (12.0)	
*Urological*	9 (5.3)	3 (3.9)	6 (6.5)	
**Dialysis vintage**, months (IQR)	37.7 (17.4–82.7)	29.1 (16.6–52.3)	50.2 (17.5–93.3)	0.009
**Dialysis type**, N (%)				0.085
*Hemodialysis*	141 (83.4)	62 (80.5)	79 (85.9)	
*Peritoneal Dialysis*	24 (14.2)	11 (14.3)	13 (14.1)	
*Preemptive*	4 (2.4)	4 (5.2)	–	
**HLA mismatch (ABDR), N (IQR)**	–	2 (1-3)	–	–
**PRA>0, N (%)**	–	15 (19.5)	–	–
**Immunosuppression**, N (%)				–
*Pred+TAC+MPS*	–	43 (55.8)	–	
*Pred+TAC+AZA*	–	32 (41.6)	–	
*Pred+TAC+mTORi*	–	2 (2.6)	–	

Abbreviations – N: number; IQR: interquartile range; CKD: Chronic Kidney Disease; HLA: Human Leukocyte Antigen; PRA: panel-reactive antibody; Pred: prednisone; TAC: tacrolimus; MPS: mycophenolate sodium; AZA: azathioprine; mTORi: mTOR inhibitor.

In the transplant group, the median donor age was 49 years, and most donors were male (58.4%) and white (53.2%). Most transplants were performed with deceased donors (80.5%), with a median KDPI of 54%, serum creatinine of 1.56 mg/dL, and 37.1% were expanded criteria donors. The median cold ischemia time was 22.2 h (Table 2). All KTRs received induction therapy with rabbit anti-thymocyte globulin, and most received prednisone, tacrolimus, and mycophenolate sodium as maintenance therapy (Table 1).

**Table 2:** Demographic characteristics of kidney donors

	Transplant N = 77
**Age**, years (IQR)	49 (34.5–59)
**Male sex**, N (%)	45 (58.4)
**Ethnicity**, N (%)	
*White*	41 (53.2)
*Mixed*	31 (40.3)
*Black*	5 (6.5)
**Hypertension**, N (%)	20 (26.0)
**Diabetes Mellitus**, N (%)	5 (6.5)
**Deceased donor**, N (%)	62 (80.5)
**KDPI**, %	54 (37–82)
**KDRI**	1.04 (0.88–1.40)
**Creatinine**, mg/dL (IQR)	1.56 (1.16–2.70)
**Expanded Criteria**, N (%)	23 (37.1)
**Cold Ischemia Time**, hours (IQR)	22.2 (17.3–28.7)

Abbreviations – N: number; IQR: interquartile range; KDPI: Kidney Donor Profile Index; KDRI: Kidney Donor Risk Index.

### Primary Vaccine Schedules and Boosters

The distribution of primary SARS-CoV-2 vaccine types is detailed in Table 3. Among all patients included, the most administered vaccine was ChAdOx1 nCoV-19, received by 81 (47.9%) patients. This primary vaccine schedule was predominant in the transplant group, accounting for 79.2% of cases (n = 61), compared to 21.7% (n = 20) in the dialysis group. Conversely, CoronaVac was the main primary vaccine schedule in the dialysis group, administered to 68 (73.9%) patients, while 8 (10.4%) KTRs received this vaccine. The BNT162b2 mRNA vaccine was used in 5.3% (n = 9) of the overall population, with a slightly higher frequency in KTRs (9.1%) than in dialysis patients (2.2%). The Ad26.COV2.S vaccine was used in 1.2% (n = 2) of patients, with one case in each group. In the dialysis group, only one patient (1.1%) received a heterologous vaccination schedule that combined the BNT162b2 and ChAdOx1 vaccines after a 3-month interval (Table 3). However, the National Immunization Program did not account for this schedule, leading to a failure during the immunization process.

**Table 3:** Primary vaccination schedule of the study population

	Total N = 169	Transplant N = 77	Dialysis N = 92	p
**Vaccine**, N (%)				<0.001
*ChAdOx1 nCoV-19*	81 (47.9)	61 (79.2)	20 (21.7)	
*CoronaVac*	76 (45.0)	8 (10.4)	68 (73.9)	
*BNT162b2*	9 (5.3)	7 (9.1)	2 (2.2)	
*Ad26.COV2.S*	2 (1.2)	1 (1.3)	1 (1.1)	
*Heterologous (BNT162b2 + ChAdOx1)*	1 (0.6)	0 (0.0)	1 (1.1)	

Abbreviation – N: number.

Dialysis patients are vulnerable to poor outcomes of COVID-19 and therefore were prioritized for vaccine boosters. Before inclusion, 139 (82.2%) patients had received one or two booster doses, 116 (68.6%) patients had received three doses, with a similar proportion between the groups (68.8% vs. 68.5%; p = 0.961), and 23 (13.6%) patients had already received four doses, with a higher proportion in the dialysis group (7.8% vs. 18.5%; p = 0.057) (Table 4). The time between the last vaccine dose received and inclusion in the study was shorter in the transplant group (96 vs. 161 days; p = 0.006). The BNT162b2 vaccine was predominantly given to KTRs, while the CoronaVac vaccine was primarily administered to patients on dialysis (Table 4).

**Table 4:** Vaccination status at study inclusion

Screening	Total N = 169	Transplant N = 77	Dialysis N = 92	p
***Doses received,** N (%)*				0.057
*3*	116 (68.6)	53 (68.8)	63 (68.5)	
*4*	23 (13.6)	6 (7.8)	17 (18.5)	
***Last vaccine received,** N (%)*				<0.001
*BNT162b2*	76 (45.0)	44 (57.1)	32 (34.8)	
*CoronaVac*	66 (39.1)	18 (23.4)	48 (52.2)	
*ChAdOx1 nCoV-19*	20 (11.8)	13 (16.9)	7 (7.6)	
*Ad26.COV2.S*	7 (4.1)	2 (2.6)	5 (5.4)	
**Last dose**, days (IQR)	115 (49–168)	96 (56–139)	161 (34–182)	0.006

Abbreviations – N: number; IQR: interquartile range.

**Figure 2:**
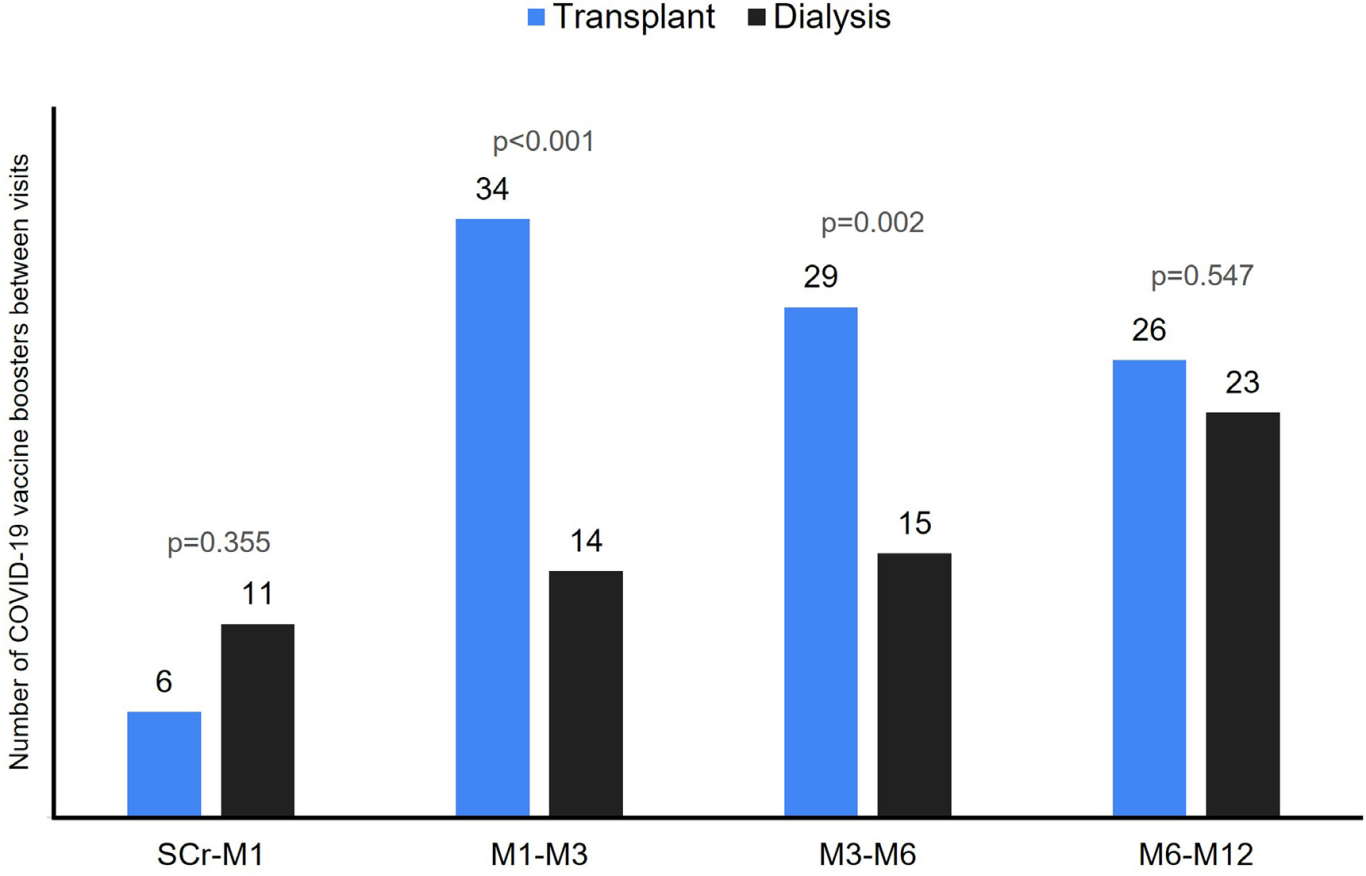
Number of COVID-19 vaccine boosters administered between study visits.

After inclusion, patients continued to receive vaccine boosters during follow-up (Figure 2). Between the screening visit and M1, KTRs and dialysis patients received a similar number of vaccine boosters (6 vs. 11; p = 0.355). Between M1 and M3 (34 vs. 14; p < 0.001) and between M3 and M6 (29 vs. 15; p = 0.002), KTRs received more booster doses, while, in the final period from M6 to M12, both groups received a similar number of doses (26 vs. 23; p = 0.547) (Figure 2). The BNT162b2 vaccine was the most frequently administered booster among KTRs throughout the study period and among dialysis patients at the M1 and M12 visits. However, during the M3 and M6 visits, dialysis patients predominantly received the Ad26.COV2.S and ChAdOx1 nCoV-19 vaccines, respectively (Table 5).

**Table 5:** Boosters received during the study

	M1			M3			M6			M12		
	Transplant N = 72	Dialysis N = 85	p	Transplant N = 70	Dialysis N = 84	p	Transplant N = 70	Dialysis N = 81	p	Transplant N = 67	Dialysis N = 68	p
**Vaccine**, N (%)			0.493			0.087			0.631			0.643
*BNT162b2*	2 (33.3)	6 (54.5)		19 (55.9)	3 (21.4)		13 (44.8)	4 (26.7)		17 (65.4)	16 (69.6)	
*ChAdOx1 nCoV-19*	1 (16.7)	1 (9.1)		4 (11.8)	1 (7.1)		7 (24.1)	6 (40.0)		0 (0.0)	1 (4.3)	
*Ad26.COV2.S*	2 (33.3)	4 (36.4)		9 (26.5)	9 (64.3)		5 (17.2)	3 (20.0)		2 (7.7)	2 (8.7)	
*CoronaVac*	1 (16.7)	0 (0.0)		2 (5.9)	1 (7.1)		4 (13.8)	2 (13.3)		7 (26.9)	4 (17.4)	
**Last dose** *, days (IQR)	118 (75–164)	146 (51–210)	0.117	67 (46–147)	138 (89–275)	<0.001	110 (68–141)	195 (122–343)	<0.001	207 (93–291)	288 (96–522)	0.012

Abbreviations – M: month; N: number; IQR: interquartile range.

Notes – *Days since the last dose received until the study visit.

### Cellular Immunity

**Figure 3:**
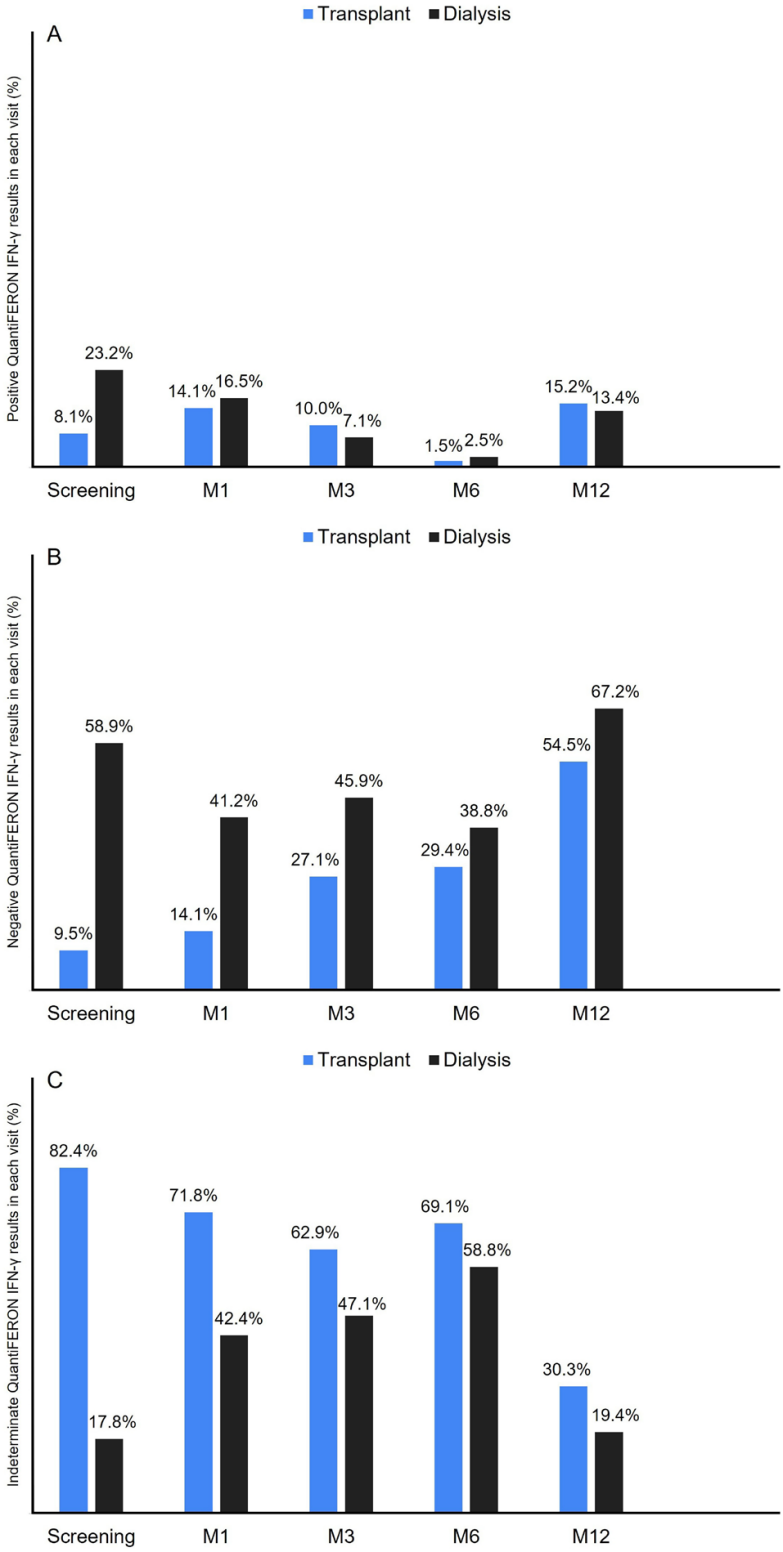
Proportion of positive (A), negative (B), and indeterminate (C) QuantiFERON IFN-γ responses by study group and visit.

Cellular immunity was evaluated by IGRA at study inclusion and at 1, 3, 6, and 12 months (Figure 3).

Positive results were observed predominantly in patients in the dialysis group only during the screening visit (8.1% vs. 23.2%), remaining similar at following visits (Figure 3). Negative results were more frequent in the dialysis group at screening (9.5% vs. 58.9%), M1 (14.1% vs. 41.2%), and M3 (27.1% vs. 45.9%), showing similar results at subsequent visits (Figure 3). Indeterminate results were more common among KTRs at screening (82.4% vs. 17.8%), M1 (71.8% vs. 42.4%), and M3 (62.9% vs. 47.1%), with comparable rates at the last two visits (Figure 3). Over the 12 months of follow-up, there was a generally low proportion of positive results, along with a trend toward increasing negative results. Both groups exhibited a high rate of indeterminate results at all study visits, particularly among KTRs.

Over time, KTRs showed a gradual increase in negative results (9.5%, 14.1%, 27.1%, 29.4%, and 54.5%) and a decrease in indeterminate results (82.4%, 71.8%, 62.9%, 69.1%, and 30.3%) and positive results (8.1%, 14.1%, 10.0%, 1.5%, and 15.2%), except at the M12 visit. Dialysis patients showed a more variable pattern during follow-up (Figure 3).

## Discussion

This study is a *post-hoc* analysis of a previously published prospective, observational, single-center clinical investigation evaluating the immune response kinetics to SARS-CoV-2 vaccination in patients with end-stage CKD^5^. This *post-hoc* analysis was designed to evaluate the kinetics of the cellular immune response after SARS-CoV-2 vaccination, while the original study addressed broader clinical aspects and humoral response outcomes^5^. Here, we specifically assessed the kinetics of the cellular immune response in patients undergoing kidney transplantation and compared them to those who remained on dialysis, excluding from the analyses those who developed COVID-19 during the follow-up.

Our findings demonstrated a high proportion of indeterminate QuantiFERON results in both groups, particularly among KTRs, which limits the interpretation of cellular immunity in this population. Furthermore, we could not establish a unique pattern of cellular response kinetics. Similar limitations have been reported in other studies involving transplant and dialysis patients, which may explain this pattern. Contributing factors include lymphopenia, T-cell dysfunction, and the use of T-cell-depleting agents, notably rabbit anti-thymocyte globulin, which impair test reliability^14-16^. Studies using the QuantiFERON test for tuberculosis have shown an incidence of indeterminate results ranging from 7% to 28%. This issue is particularly pronounced in solid organ or hematologic transplant recipients and individuals living with HIV who have lymphopenia^17,18,19^.

In hemodialysis patients, the test’s performance remains consistent, regardless of whether the sample is collected immediately before or after dialysis^20^. However, immune dysfunction caused by uremia in dialysis patients, along with the chronic use of immunosuppressive drugs by KTRs, can affect the release of IFN-γ by lymphocytes^21^. Remarkably, a positive cellular immune response is more frequently observed in dialysis patients compared to KTRs, which may explain historical differences in lethality between these groups^2,21^. Additionally, the QuantiFERON test faces challenges in its pre-analytical execution that complicate the interpretation of results. These challenges include the need for careful collection, incubation, and centrifugation techniques. To address these issues, our research team delegated and trained specific professionals for each stage of the test to ensure accuracy and reliability.

In our cohort, KTRs received a higher number of SARS-CoV-2 vaccine boosters than dialysis patients, reflecting efforts to compensate for their lower immunogenicity. However, the QuantiFERON test results showed no clear trend toward a positive cellular response, reinforcing the challenge of assessing vaccine-induced cellular immunogenicity in patients with chronic immunosuppressive use. In addition, the heterogeneity in primary and booster vaccine schedules, as well as the use of different vaccine platforms (viral vector, inactivated virus, and mRNA), did not seem to influence the cellular immune response or its kinetics in either group.

A few studies have longitudinally assessed cellular immunity after vaccination in dialysis patients and transplant recipients, and the available data show considerable methodological heterogeneity^21^. Recent studies have highlighted the significance of cellular immunity in protecting against severe SARS-CoV-2 infection, even in the absence of robust humoral responses^21^. T-cell-mediated response is necessary for generating and maintaining neutralizing antibody titers against SARS-CoV-2, and it also plays a critical role in reducing viremia and disease severity, even in individuals who do not seroconvert or have poor humoral responses, as is often seen in KTRs and dialysis patients^21^. A systematic review and meta-analysis of 45 studies evaluating cellular immune responses to SARS-CoV-2 vaccination in KTRs, dialysis patients, and the general population demonstrated that the cellular immune response in KTRs was lower than that in dialysis patients (OR 0.24; 95% CI 0.16–0.34) and that in the general population (OR 0.10; 95% CI 0.07–0.14)^21^. Despite the use of different assays (ELISpot, IGRA, and flow cytometry) and variability in vaccine types and dosing intervals, the results were consistent in showing the poor cellular response in KTRs and its association with immunosuppression (mycophenolate mofetil, triple regimens, and shorter time since transplantation)^21^.

These patients face a higher COVID-19-related mortality rate than the general population due to a greater burden of comorbidities and reduced immunogenicity, which is influenced both by the uremic status and the chronic use of immunosuppressive drugs^22,23^. This immunological lethargy contributes to an impaired vaccine response, and even among those who achieve seroconversion, there is a progressive decline in humoral and cellular immune kinetics as CKD advances^4^. Our findings reinforce the importance of individual protective measures, such as mask-wearing, and collective strategies, such as mass vaccination campaigns. Periodic vaccine boosters for high-risk populations continue to be strongly recommended to prevent severe disease, long-term complications, and death.

Finally, this study has some limitations. Since patients were included during the critical phase of the Omicron variant of the COVID-19 pandemic between December 2021 and May 2022, we could not control the timing of boosters or the type of vaccine administered. Given the public health emergency, it was neither ethical nor feasible to include unvaccinated controls or to standardize vaccine type and booster intervals. Vaccine allocation followed national distribution policies and depended on global supply availability. Moreover, transplant recipients under chronic immunosuppression and dialysis patients represented a particularly high-risk group, precluding any deviation from recommended vaccination schedules. Although the number of participants analyzed was reduced, mainly due to the exclusion of patients who developed COVID-19 during follow-up, this approach was necessary to avoid the confounding effect of natural infection on vaccine-induced immune responses. As viral infection strongly influences post-vaccination immunogenicity, excluding these cases helped to ensure a more accurate interpretation of the cellular response kinetics. Considering these points, the comparison of cellular immune responses between different vaccine types was not feasible and should be interpreted with caution. In addition, the IFN-γ release test QuantiFERON demonstrated a high number of indeterminate results and variable kinetics during follow-up, particularly among KTRs, suggesting the need for cautious interpretation of the results. This finding likely reflects both technical difficulties inherent to the assay and patient-related factors. The QuantiFERON assay requires precise handling conditions, including immediate incubation and controlled centrifugation, which can affect test performance. Furthermore, T-cell depletion induced by anti-thymocyte globulin, persistent lymphopenia, and functional T-cell impairment caused by chronic immunosuppression may contribute to reduced IFN-γ release and, consequently, to indeterminate results^14,15,16^.

Future studies should explore complementary assays, such as ELISpot and flow cytometry, which can more accurately assess SARS-CoV-2–specific cellular immunity in immunosuppressed patients. These methods quantify antigen-specific T-cell responses and characterize functional subsets, offering greater sensitivity than IGRA. However, each assay captures distinct aspects of T-cell activity, and no single test fully reflects the complexity of cellular immunity. Recent evidence supports combining multiple assays to improve the evaluation of vaccine-induced responses in kidney transplant and dialysis patients^21^.

From a clinical standpoint, our findings support the need for tailored vaccination strategies in patients with advanced kidney disease, particularly transplant recipients under chronic immunosuppression. The limited and heterogeneous cellular responses observed in this study suggest that standard vaccination schedules may be insufficient to achieve durable protection in these populations. Regular humoral and cellular monitoring of immune responses could help identify non-responders and guide personalized booster regimens. National vaccination policies should consider prioritizing these high-risk groups for additional or adjuvanted vaccine doses, as well as early antiviral access in case of breakthrough infection. Such measures may optimize protection and reduce the risk of severe COVID-19 outcomes among dialysis and transplant patients.

## Conclusion

Our findings indicate that using the QuantiFERON test to evaluate SARS-CoV-2 vaccine-induced cellular immunity has significant limitations for kidney transplant recipients and dialysis patients. This is particularly evident due to the high number of indeterminate results observed in these groups. Although transplant patients received more vaccine boosters than dialysis patients, we could not identify a clear and sustained cellular immune response, reflecting the effects of immunosuppression on T-cell function. These results underscore the challenges of monitoring cellular immunity in immunocompromised populations and highlight the need for more sensitive and reliable methods to assess protective immunity in high-risk groups, such as ELISpot or flow cytometry. A regular schedule of vaccine boosters is necessary to reduce the risk of severe disease and mortality in this vulnerable population.

## Data Availability

The datasets generated and/or analyzed during the current study are available from the corresponding author upon reasonable request.
