# Supplementary material for: Kinetics of cellular immune response to SARS-CoV-2 vaccine boosters in dialysis and kidney transplant patients without infection
Source: J Bras Nefrol. 2026 Mar 23;48(1):e20250206. doi: 10.1590/2175-8239-JBN-2025-0206en (PMC13008128; doi:10.1590/2175-8239-JBN-2025-0206en)
Supplement: Translation

# Translation: Cinética da resposta imune celular à vacina contra SARS-CoV-2 em diálise e transplante renal sem infecção

**DOI:** 10.1590/2175-8239-JBN-2025-0206pt

**Authors:** Roberto Matias Souza, Julia Soares Reis, Helio Tedesco-Silva, Lúcio Requião-Moura, José Medina-Pestana, Renato Demarchi Foresto

**Affiliations:** 1 Universidade Federal de São Paulo, Departamento de Medicina, Disciplina de Nefrologia, São Paulo, SP, Brasil.; 2 Hospital do Rim, Fundação Oswaldo Ramos, São Paulo, SP, Brasil.

**Keywords:** Imunidade Celular, SARS-CoV-2, Transplante de Rim, COVID-19, Vacinas, Diálise

## Abstract

**Introdução:**

Pacientes em diálise e receptores de transplante renal (RTR) apresentam resposta imune comprometida às vacinas contra a COVID-19, e a cinética imune celular após reforços vacinais é incerta. Este estudo avalia a cinética imune celular ao SARS-CoV-2 em pacientes em diálise e RTR vacinados, sem infecção prévia.

**Métodos:**

Análise *post hoc* de estudo prospectivo, observacional, de centro único, que comparou a cinética imune celular após vacinação contra SARS-CoV-2 em RTR (n = 77) e pacientes em diálise (n = 92), sem infecção prévia. A resposta de linfócitos T específicos para SARS-CoV-2, por meio do teste IGRA (QuantiFERON SARS-CoV-2 RUO), foi analisada em sangue periférico na triagem e após 1, 3, 6 e 12 meses. Comparações entre grupos foram realizadas com os testes qui-quadrado e Mann-Whitney.

**Resultados:**

Resultados IGRA positivos foram pouco frequentes em RTR e em pacientes em diálise (triagem: 8,1% vs. 23,2%; M1: 14,1% vs. 16,5%; M3: 10% vs. 7,1%; M6: 1,5% vs. 2,5%; M12: 15,2% vs. 13,4%), mas resultados indeterminados foram comuns (triagem: 82,4% vs. 17,8%; M1: 71,8% vs. 42,4%; M3: 62,9% vs. 47,1%; M6: 69,1% vs. 58,8%; M12: 30,3% vs. 19,4%), especialmente entre RTR, embora tenham recebido mais doses de reforço (95 vs. 63; p < 0,001). Não houve correlação entre número de doses de vacina e positividade do IGRA.

**Conclusão:**

Apesar do uso de doses de reforço, pacientes em diálise e RTR apresentaram resposta imune celular limitada e variável, destacando a necessidade de estratégias aprimoradas de vacinação e monitorização nessas populações.

## Introdução

A pandemia de COVID-19 levou a milhões de óbitos em todo o mundo, sendo o Brasil um dos países mais afetados. Esse cenário representou um risco significativo para receptores de transplante renal (RTR), que apresentaram taxa de letalidade superior a 24%, em comparação com aproximadamente 3% na população geral no início da pandemia^1^.

Apesar dos esforços contínuos para desenvolver vacinas eficazes e seguras, pacientes em diálise e RTR continuaram a apresentar altas taxas de mortalidade mesmo após a vacinação^1,2^. Apenas após a ampla aplicação de doses de reforço e o surgimento da variante Ômicron houve melhora dos desfechos da doença nessas populações^3^.

Após a vacinação, RTR exibem resposta imune celular e humoral mais fraca do que a população geral e pacientes em outros estágios de Doença Renal Crônica (DRC)^4^. Essa situação levantou preocupações sobre a segurança da realização de transplantes durante a pandemia e sobre se as altas taxas de letalidade, inclusive em indivíduos mais jovens e com menos comorbidades, estariam relacionadas ao regime imunossupressor ao qual são submetidos^1^.

Grande parte da atenção tem sido voltada à cinética da resposta imune humoral adquirida pela vacinação contra o SARS-CoV-2 em RTR^5,6^. Sabe-se que a resposta humoral é atenuada em RTR devido ao uso de drogas imunossupressoras, que reduzem o desenvolvimento de uma resposta imune eficiente contra o patógeno, como a inibição da ativação de linfócitos e da interação com células apresentadoras de antígeno, além da redução das respostas de células B de memória^7^. Em comparação com pacientes com doença renal em estágio avançado que permaneceram em diálise, RTR apresentaram títulos de IgG anti-SARS-CoV-2 mais baixos ao longo do tempo, mesmo recebendo mais doses de reforço, além de maior incidência de COVID-19^5^. No entanto, a resposta imune celular após a vacinação é um aspecto importante a ser considerado nos mecanismos de defesa imune contra patógenos, mas tem sido pouco explorada no contexto da infecção por SARS-CoV-2. Essa resposta já demonstrou ser efetiva tanto em RTR quanto em indivíduos saudáveis, conferindo proteção contra a doença mesmo em situações nas quais o componente humoral da resposta imune não foi proeminente^8,9^.

Assim, este estudo tem como objetivo avaliar o impacto da imunossupressão do transplante renal na cinética da resposta imune celular adquirida por meio da vacinação contra o SARS-CoV-2 em pacientes que não desenvolveram COVID-19. Este estudo foi conduzido exclusivamente em pacientes adultos, e os achados podem não ser generalizáveis para populações pediátricas.

## Métodos

### Desenho do Estudo e População

Trata-se de uma análise *post hoc* de um estudo prospectivo, observacional, de centro único, previamente publicado, que comparou a cinética da resposta imune adquirida pela vacinação contra o SARS-CoV-2 em pacientes submetidos ao transplante renal e em pacientes que permaneceram em diálise^5^. A coorte previamente publicada investigou a cinética da resposta imune humoral à vacinação contra o SARS-CoV-2 em RTR e em pacientes em diálise^5^. Nesta análise atual, buscamos avaliar o efeito da imunossupressão do transplante renal, focando exclusivamente na cinética da resposta imune celular, avaliada por IGRA, adquirida por meio da vacinação contra o SARS-CoV-2 em pacientes que não desenvolveram COVID-19. Esse desenho permitiu isolar a resposta de células T induzida pela vacina, sem a influência confundidora de infecção natural (Figura 1).

**Figura 1:** Fluxograma da população do estudo.

Foram incluídos pacientes adultos com mais de 18 anos de idade, com DRC em estágio avançado, completamente vacinados, que tinham soroconvertido e não apresentavam diagnóstico prévio de COVID-19. Todos os participantes estavam em lista de espera para transplante renal e ambas as coortes foram recrutadas de forma concomitante durante o período do estudo. Pacientes que foram submetidos ao transplante foram alocados no grupo transplante, enquanto aqueles que permaneceram em diálise foram alocados no grupo diálise (Figura 1). O grupo transplante incluiu receptores de rins de doadores vivos e falecidos. Os critérios de exclusão incluíram pacientes com resultado negativo para IgG anti-SARS-CoV-2 na inclusão, indivíduos vivendo com HIV e pacientes em tratamento oncológico. O período do estudo, durante a pandemia, foi marcado pela predominância de circulação da variante Ômicron do SARS-CoV-2, em um contexto de alta cobertura vacinal na população. O *Kidney Donor Profile Index* é um escore que resume a qualidade esperada de um rim de doador falecido com base em características do doador.

### Procedimentos do Estudo

Este estudo foi aprovado pelo Comitê de Ética em Pesquisa da Universidade Federal de São Paulo (parecer nº 5.156.043). Todos os participantes elegíveis assinaram termo de consentimento livre e esclarecido antes da inclusão. O estudo seguiu os princípios da Declaração de Helsinque e esteve em conformidade com as diretrizes de Boas Práticas Clínicas. Cada paciente foi acompanhado por 12 meses a partir da data de inclusão, com coleta de amostras de sangue logo após a inclusão e subsequentemente, em 1, 3, 6 e 12 meses para avaliação da resposta imune ao SARS-CoV-2 (Figura 1). Para os participantes do grupo transplante, a amostra inicial de sangue foi obtida antes do início da terapia imunossupressora. O estudo não modificou os esquemas imunossupressores de rotina utilizados no transplante renal. Também não foram feitas recomendações sobre vacinação ou sobre o momento das doses adicionais de reforço. Todos os participantes receberam vacinas contra o SARS-CoV-2 pelo sistema público de saúde, conforme as diretrizes do Ministério da Saúde. Todos os transplantes renais e o seguimento dos pacientes em diálise incluídos no estudo foram realizados em nosso centro.

### Avaliação da Imunidade Celular

A resposta imune celular foi avaliada por detecção qualitativa de IFN-gama produzido por respostas de linfócitos T CD4+ e CD8+ a peptídeos do SARS-CoV-2, utilizando o kit *QuantiFERON SARS-CoV-2 RUO Starter Set (QIAGEN)*. O ponto de corte para positividade foi definido como ≥0,35 IU/mL. As amostras de sangue foram coletadas em três tubos distintos: (1) Tubo Nil, contendo apenas heparina e utilizado como controle negativo; (2) Tubo Ag3, contendo antígenos específicos do SARS-CoV-2 para estimular a resposta imune; e (3) Tubo Mitógeno, contendo fito-hemaglutinina como controle positivo^10^. Após a coleta, os tubos foram homogeneizados e incubados a 37 ± 1 °C por 16 horas. Em seguida, as amostras foram centrifugadas a 3.000 rpm por 15 minutos para separação do plasma, que foi armazenado a -70 °C para análise posterior. A quantificação dos níveis de IFN-γ no plasma foi realizada por ELISA. O ELISA QuantiFERON SARS-CoV-2 utiliza um padrão de IFN-γ humano recombinante, calibrado contra uma preparação de referência (NIH Ref: Gxg01-902-535). Os resultados do teste foram expressos em IU/mL, com base em uma curva padrão gerada a partir de diluições seriadas do padrão de referência. O Tubo Mitógeno foi utilizado como controle positivo para confirmar a responsividade da amostra, enquanto o Tubo Nil permitiu o ajuste para a produção basal de IFN-γ^10^.

### Vacinas Administradas e o Programa Nacional de Imunizações

A campanha nacional de vacinação contra a COVID-19 no Brasil teve início em 17 de janeiro de 2021, com a administração da CoronaVac, uma vacina de vírus inativado^11^. O processo de vacinação priorizou inicialmente grupos de alto risco. Além da CoronaVac, outras vacinas foram disponibilizadas e distribuídas pelo Programa Nacional de Imunizações: ChAdOx1 nCoV-19 (AstraZeneca), baseada em plataforma de vetor viral; BNT162b2 (Comirnaty, Pfizer-BioNTech), que utiliza tecnologia de RNA mensageiro; e Ad26.COV2.S (Janssen), também baseada em vetor viral^12,13^.

### Protocolo Institucional de Imunossupressão

O protocolo imunossupressor institucional inclui terapias de indução e de manutenção. No Hospital do Rim, todos os receptores de transplante renal recebem 1.000 mg de metilprednisolona no intraoperatório, seguida de uma dose única de 3 mg/kg de globulina antitimócito de coelho administrada no dia seguinte à cirurgia. A imunossupressão de manutenção é baseada na combinação de um corticosteroide, um inibidor de calcineurina e um terceiro agente, azatioprina ou micofenolato de sódio, escolhidos conforme o perfil de risco imunológico do receptor e o tipo de doador.

### Análise Estatística

As variáveis qualitativas foram resumidas em distribuições de frequência, expressas em números absolutos e porcentagens, e os grupos foram comparados utilizando-se os testes do qui-quadrado ou exato de Fisher. O teste de normalidade de Kolmogorov-Smirnov foi utilizado para verificar se a distribuição de probabilidade associada ao conjunto de variáveis quantitativas poderia ser aproximada pela distribuição normal. As variáveis quantitativas foram apresentadas como mediana e intervalo interquartil para variáveis não paramétricas e como média e desvio padrão para variáveis paramétricas. As diferenças entre os grupos foram analisadas com o teste de Mann-Whitney.

Todas as análises estatísticas foram realizadas utilizando o *Statistical Package for the Social Sciences* (SPSS) versão 29 (*IBM Corp., Released 2022. IBM SPSS Statistics for Windows, Version 29.0, Armonk, NY: IBM Corp*.). Valores de p inferiores a 0,05 foram considerados estatisticamente significativos, com intervalo de confiança de 95%.

## Resultados

### Demografia

Um total de 221 pacientes foi incluído no estudo original, 113 no grupo transplante (Tx) e 108 no grupo diálise. Durante os 12 meses de seguimento, 36 RTR e 16 pacientes em diálise desenvolveram COVID-19 e foram censurados, resultando em 77 pacientes no grupo transplante e 92 no grupo diálise incluídos nesta análise (Figura 1).

A mediana da idade dos receptores (45,4 vs. 54,0 anos; p < 0,001) e o tempo em diálise (29,1 vs. 50,2 meses; p < 0,009) foram maiores no grupo diálise. As demais características demográficas foram semelhantes entre os grupos e estão resumidas na Tabela 1. Em ambos os grupos, a maioria dos pacientes era do sexo masculino (54,4% vs. 58,7%; p = 0,588), branca (59,7% vs. 57,6%; p = 0,763), hipertensa (83,1% vs. 87,0%; p = 0,484), diabética (19,5% vs. 27,2%; p = 0,241), com etiologia de DRC indeterminada (40,3% vs. 28,3%; p = 0,111) e realizava hemodiálise como terapia renal substitutiva (80,5% vs. 85,9%; p = 0,085) (Tabela 1).

**Tabela 1:** Características demográficas

	Total N = 169	TransplanteN = 77	DiáliseN = 92	p
**Idade**, anos (IIQ)	50,4 (39,2–59,4)	45,4 (36,1–57,0)	54,0 (45,4–63,0)	<0,001
**Sexo masculino**, N (%)	96 (56,8)	42 (54,5)	54 (58,7)	0,588
**Raça**, N (%)				0,763
*Branca*	99 (58,6)	46 (59,7)	53 (57,6)	
*Parda*	49 (29,0)	23 (29,9)	26 (28,3)	
*Negra*	21 (12,4)	8 (10,4)	13 (14,1)	
**Hipertensão**, N (%)	144 (85,2)	64 (83,1)	80 (87,0)	0,484
**Diabetes Mellitus**, N (%)	40 (23,7)	15 (19,5)	25 (27,2)	0,241
**Etiologia da DRC**, N (%)				0,111
*Indeterminada*	57 (33,7)	31 (40,3)	26 (28,3)	
*Glomerulonefrite*	41 (24,3)	22 (28,6)	19 (20,7)	
*Diabetes Mellitus*	30 (17,8)	13 (16,9)	17 (18,5)	
*Hipertensão*	17 (10,1)	4 (5,2)	13 (14,1)	
*Doença Renal Policística*	15 (8,9)	4 (5,2)	11 (12,0)	
*Urológica*	9 (5,3)	3 (3,9)	6 (6,5)	
**Tempo em diálise**, meses (IIQ)	37,7 (17,4-82,7)	29,1 (16,6-52,3)	50,2 (17,5-93,3)	0,009
**Terapia Renal Substitutiva**, N (%)				0,085
*Hemodiálise*	141 (83,4)	62 (80,5)	79 (85,9)	
*Diálise Peritoneal*	24 (14,2)	11 (14,3)	13 (14,1)	
*Preemptivo*	4 (2,4)	4 (5,2)	–	
***Mismatches*** **HLA (ABDR), N (IIQ)**	–	2 (1-3)	–	–
**PRA>0%, N (%)**	–	15 (19,5)	–	–
**Imunossupressão**, N (%)				–
*Pred+TAC+MPS*	–	43 (55,8)	–	
*Pred+TAC+AZA*	–	32 (41,6)	–	
*Pred+TAC+imTOR*	–	2 (2,6)	–	

Abreviaturas – N: número; IIQ: interval interquartil; DRC: doença renal crônica; HLA: *Human Leukocyte Antigen*; PRA: *panel-reactive antibody*; Pred: prednisona; TAC: tacrolimo; MPS: micofenolato de sódio; AZA: azatioprina; mTORi: inibidor da mTOR.

No grupo transplante, a mediana de idade dos doadores foi de 49 anos, a maioria era do sexo masculino (58,4%) e de raça branca (53,2%). A maior parte dos transplantes foi realizada com doadores falecidos (80,5%), com mediana de KDPI de 54%, creatinina sérica de 1,56 mg/dL, e 37,1% foram doadores de critérios expandidos. A mediana do tempo de isquemia fria foi de 22,2 horas (Tabela 2). Todos os RTR receberam terapia de indução com globulina antitimócito, e a maioria recebeu prednisona, tacrolimo e micofenolato de sódio como terapia de manutenção (Tabela 1).

**Tabela 2:** Características demográficas dos doadores

	Transplante N = 77
**Idade**, anos (IIQ)	49 (34,5–59)
**Sexo masculino**, N (%)	45 (58,4)
**Raça**, N (%)	
*Branca*	41 (53,2)
*Parda*	31 (40,3)
*Negra*	5 (6,5)
**Hipertensão**, N (%)	20 (26,0)
**Diabetes Mellitus**, N (%)	5 (6,5)
**Doador falecido**, N (%)	62 (80,5)
**KDPI**, %	54 (37–82)
**KDRI**	1,04 (0,88–1,40)
**Creatinina final**, mg/dL (IIQ)	1,56 (1,16–2,70)
**Critério expandido**, N (%)	23 (37,1)
**Tempo de Isquemia Fria**, horas (IIQ)	22,2 (17,3–28,7)

Abreviaturas – N: número; IIQ: interval interquartil; KDPI: *Kidney Donor Profile Index*; KDRI: *Kidney Donor Risk Index*.

### Esquemas Vacinais Primários e Doses de Reforço

A distribuição dos tipos de vacinas primárias contra o SARS-CoV-2 está detalhada na Tabela 3. Entre todos os pacientes incluídos, a vacina mais administrada foi a ChAdOx1 nCoV-19, recebida por 81 pacientes (47,9%). Esse esquema vacinal primário foi predominante no grupo transplante, correspondendo a 79,2% dos casos (n = 61), em comparação com 21,7% (n = 20) no grupo diálise. Por outro lado, a CoronaVac foi o principal esquema vacinal primário no grupo diálise, administrada a 68 pacientes (73,9%), enquanto apenas 8 RTR (10,4%) a receberam. A vacina de mRNA BNT162b2 foi utilizada em 9 pacientes (5,3%) do total da população, com frequência ligeiramente maior em RTR (9,1%) do que em pacientes em diálise (2,2%). A vacina Ad26.COV2.S foi utilizada em 2 pacientes (1,2%), com um caso em cada grupo. No grupo diálise, apenas um paciente (1,1%) recebeu um esquema vacinal heterólogo que combinou as vacinas BNT162b2 e ChAdOx1 após intervalo de 3 meses (Tabela 3). No entanto, esse esquema não foi reconhecido pelo Programa Nacional de Imunizações, o que levou a uma falha no processo de imunização.

**Tabela 3:** Esquema vacinal primário da população do estudo

	Total N = 169	Transplante N = 77	Diálise N = 92	p
**Vacina**, N (%)				<0,001
*ChAdOx1 nCoV-19*	81 (47,9)	61 (79,2)	20 (21,7)	
*CoronaVac*	76 (45,0)	8 (10,4)	68 (73,9)	
*BNT162b2*	9 (5,3)	7 (9,1)	2 (2,2)	
*Ad26.COV2.S*	2 (1,2)	1 (1,3)	1 (1,1)	
*Heterólogo (BNT162b2 + ChAdOx1)*	1 (0,6)	0 (0,0)	1 (1,1)	

Abreviatura – N: número.

Pacientes em diálise são vulneráveis aos desfechos desfavoráveis de COVID-19, por isso foram priorizados para receber doses de reforço. Antes da inclusão, 139 pacientes (82,2%) tinham recebido uma ou duas doses de reforço, 116 (68,6%) haviam recebido três doses, com proporção semelhante entre os grupos (68,8% vs. 68,5%; p = 0,961), e 23 pacientes (13,6%) já haviam recebido quatro doses, com maior proporção no grupo diálise (7,8% vs. 18,5%; p = 0,057) (Tabela 4). O intervalo entre a última dose de vacina recebida e a inclusão no estudo foi menor no grupo transplante (96 vs. 161 dias; p = 0,006). A vacina BNT162b2 foi predominantemente aplicada em RTR, enquanto a CoronaVac foi administrada principalmente em pacientes em diálise (Tabela 4).

**Tabela 4:** Situação vacinal na inclusão do estudo (visita de triagem)

Triagem	Total N = 169	Transplante N = 77	Diálise N = 92	p
***Doses recebidas***, N (%)				0,057
*3*	116 (68,6)	53 (68,8)	63 (68,5)	
*4*	23 (13,6)	6 (7,8)	17 (18,5)	
***Última vacina recebida***, N (%)				<0,001
*BNT162b2*	76 (45,0)	44 (57,1)	32 (34,8)	
*CoronaVac*	66 (39,1)	18 (23,4)	48 (52,2)	
*ChAdOx1 nCoV-19*	20 (11,8)	13 (16,9)	7 (7,6)	
*Ad26.COV2.S*	7 (4,1)	2 (2,6)	5 (5,4)	
**Última dose**, dias (IIQ)	115 (49–168)	96 (56–139)	161 (34–182)	0,006

Abreviaturas – N: número; IIQ: intervalo interquartil.

Após a inclusão, os pacientes continuaram a receber doses de reforço durante o seguimento (Figura 2). Entre a visita de triagem e M1, RTR e pacientes em diálise receberam número semelhante de doses de reforço (6 vs. 11; p = 0,355). Entre M1 e M3 (34 vs. 14; p < 0,001) e entre M3 e M6 (29 vs. 15; p = 0,002), RTR receberam mais doses, enquanto, no último período, de M6 a M12, ambos os grupos receberam número semelhante de doses (26 vs. 23; p = 0,547) (Figura 2). A vacina BNT162b2 foi a dose de reforço mais administrada entre os RTR ao longo de todo o estudo e entre os pacientes em diálise nas visitas M1 e M12. No entanto, nas visitas M3 e M6, pacientes em diálise receberam predominantemente as vacinas Ad26.COV2.S e ChAdOx1 CoV-19, respectivamente (Tabela 5).

**Figura 2:** Número de doses de reforço da vacina contra a COVID-19 administrada entre as visitas do estudo.

**Tabela 5:** Doses de reforço recebidas durante o estudo

	M1			M3			M6			M12		
	Transplante N = 72	Diálise N = 85	p	Transplante N = 70	Diálise N = 84	p	Transplante N = 70	Diálise N = 81	p	Transplante N = 67	Diálise N = 68	p
**Vacina**, N (%)			0,493			0,087			0,631			0,643
*BNT162b2*	2 (33,3)	6 (54,5)		19 (55,9)	3 (21,4)		13 (44,8)	4 (26,7)		17 (65,4)	16 (69,6)	
*ChAdOx1 nCoV-19*	1 (16,7)	1 (9,1)		4 (11,8)	1 (7,1)		7 (24,1)	6 (40,0)		0 (0,0)	1 (4,3)	
*Ad26.COV2.S*	2 (33,3)	4 (36,4)		9 (26,5)	9 (64,3)		5 (17,2)	3 (20,0)		2 (7,7)	2 (8,7)	
*CoronaVac*	1 (16,7)	0 (0,0)		2 (5,9)	1 (7,1)		4 (13,8)	2 (13,3)		7 (26,9)	4 (17,4)	
**Última dose** *, dias (IIQ)	118 (75–164)	146 (51–210)	0,117	67 (46–147)	138 (89–275)	<0,001	110 (68–141)	195 (122–343)	<0,001	207 (93–291)	288 (96–522)	0,012

Abreviaturas – M: mês; N: número; IIQ: intervalo interquartil.

Notas – *Dias desde a última dose recebida até a visita do estudo.

### Imunidade Celular

A imunidade celular foi avaliada por IGRA na inclusão do estudo e em 1, 3, 6 e 12 meses (Figura 3).

**Figura 3:** Proporção de resultados positivos (A), negativos (B) e indeterminados (C) das respostas de IFN-γ no QuantiFERON, por grupo e por visita do estudo.

Resultados positivos foram observados predominantemente em pacientes do grupo diálise apenas na visita de triagem (8,1% vs. 23,2%), mantendo-se semelhantes entre os grupos nas visitas subsequentes (Figura 3). Já os resultados negativos foram mais frequentes no grupo diálise na triagem (9,5% vs. 58,9%), em M1 (14,1% vs. 41,2%) e em M3 (27,1% vs. 45,9%), mostrando resultados semelhantes nas visitas posteriores (Figura 3). Por sua vez, os resultados indeterminados foram mais comuns entre os RTR na triagem (82,4% vs. 17,8%), em M1 (71,8% vs. 42,4%) e em M3 (62,9% vs. 47,1%), com taxas comparáveis nas duas últimas visitas (Figura 3). Ao longo dos 12 meses de seguimento, observou-se proporção geralmente baixa de resultados positivos, com tendência de aumento de resultados negativos. Ambos os grupos exibiram alta taxa de resultados indeterminados em todas as visitas do estudo, especialmente entre os RTR.

Ao longo do tempo, os RTR apresentaram aumento gradual dos resultados negativos (9,5%, 14,1%, 27,1%, 29,4% e 54,5%) e redução dos resultados indeterminados (82,4%, 71,8%, 62,9%, 69,1% e 30,3%) e positivos (8,1%, 14,1%, 10,0%, 1,5% e 15,2%), exceto na visita M12. Os pacientes em diálise apresentaram padrão mais variável de resultados durante o seguimento (Figura 3).

## Discussão

Esta é uma análise *post hoc* de um estudo clínico prospectivo, observacional, de centro único, previamente publicado, que avaliou a cinética da resposta imune à vacinação contra o SARS-CoV-2 em pacientes com DRC em estágio avançado^5^. Esta análise foi desenhada para avaliar a cinética da resposta imune celular após a vacinação contra o SARS-CoV-2, enquanto o estudo original abordou aspectos clínicos mais amplos e desfechos relacionados à resposta humoral^5^. Aqui, avaliamos especificamente a cinética da resposta imune celular em pacientes submetidos a transplante renal em comparação àqueles que permaneceram em diálise, excluindo da análise aqueles que desenvolveram COVID-19 durante o seguimento.

Nossos achados demonstraram alta proporção de resultados indeterminados no QuantiFERON em ambos os grupos, particularmente entre RTR, o que limita a interpretação da imunidade celular nessa população. Além disso, não foi possível estabelecer um padrão único de cinética da resposta celular. Limitações semelhantes foram relatadas em outros estudos envolvendo pacientes transplantados e em diálise, o que pode explicar esse padrão. Fatores contribuintes incluem linfopenia, disfunção de linfócitos T e uso de agentes depletores de células T, especialmente globulina antitimócito, que comprometem a confiabilidade do teste^14-16^. Estudos que utilizaram o QuantiFERON para tuberculose mostraram incidência de resultados indeterminados variando de 7% a 28%. Essa questão é particularmente relevante em receptores de transplantes de órgão sólido ou hematopoiético e em indivíduos vivendo com HIV com linfopenia^17-19^.

Em pacientes em hemodiálise, o desempenho do teste permanece consistente independentemente de a coleta ser realizada imediatamente antes ou após a sessão de diálise^20^. No entanto, a disfunção imune causada pela uremia em pacientes em diálise, associada ao uso crônico de imunossupressores em RTR, pode afetar a liberação de IFN-γ pelos linfócitos^21^. De forma marcante, uma resposta imune celular positiva é observada com mais frequência em pacientes em diálise do que em RTR, o que pode explicar diferenças históricas nas taxas de letalidade entre esses grupos^2,21^. Além disso, o teste QuantiFERON apresenta desafios na fase pré-analítica que dificultam a interpretação dos resultados. Esses desafios incluem necessidade de coleta, incubação e centrifugação cuidadosas. Para mitigar essas questões, nossa equipe de pesquisa delegou e treinou profissionais específicos para cada etapa do teste, com o objetivo de garantir maior acurácia e confiabilidade.

Em nossa coorte, RTR receberam número maior de doses de reforço da vacina contra o SARS-CoV-2 do que pacientes em diálise, refletindo esforços para compensar sua menor imunogenicidade. Entretanto, os resultados do QuantiFERON não evidenciaram tendência clara de aumento da resposta celular, reforçando a dificuldade de avaliar a imunogenicidade celular induzida por vacina em pacientes em uso crônico de imunossupressores. Além disso, a heterogeneidade dos esquemas vacinais primários e de reforço, assim como o uso de diferentes plataformas vacinais (vetor viral, vírus inativado e RNAm), não pareceu influenciar a resposta imune celular ou a cinética dessa resposta em nenhum dos grupos.

Poucos estudos avaliaram de forma longitudinal a imunidade celular após a vacinação em pacientes em diálise e em receptores de transplante, e os dados disponíveis demonstram considerável heterogeneidade metodológica^21^. Estudos recentes destacaram a importância da imunidade celular na proteção contra formas graves de infecção por SARS-CoV-2, mesmo na ausência de respostas humorais robustas^21^. A resposta mediada por células T é necessária para a geração e manutenção de títulos de anticorpos neutralizantes contra o SARS-CoV-2, além de desempenhar papel crítico na redução da viremia e da gravidade da doença, inclusive em indivíduos que não soroconvertem ou que apresentam resposta humoral deficiente, como frequentemente observado em RTR e em pacientes em diálise^21^. Uma revisão sistemática e metanálise de 45 estudos que avaliaram respostas imunes celulares à vacinação contra o SARS-CoV-2 em RTR, pacientes em diálise e na população geral demonstrou que a resposta imune celular em RTR foi inferior à dos pacientes em diálise (OR 0,24; IC95% 0,16-0,34) e à da população geral (OR 0,10; IC95% 0,07-0,14)^21^. Apesar do uso de diferentes ensaios (ELISpot, IGRA e citometria de fluxo) e da variabilidade dos tipos de vacinas e dos intervalos entre doses, os resultados foram consistentes em demonstrar a baixa resposta celular em RTR e sua associação com imunossupressão (micofenolato mofetil, esquemas triplos e menor tempo desde o transplante)^21^.

Esses pacientes apresentam taxas de mortalidade relacionadas à COVID-19 mais altas do que a população geral, devido à maior carga de comorbidades e à imunogenicidade reduzida, influenciada tanto pelo estado urêmico quanto pelo uso crônico de imunossupressores^22,23^. Essa letargia imunológica contribui para uma resposta vacinal prejudicada e, mesmo entre aqueles que alcançam soroconversão, há queda progressiva da cinética da resposta imune humoral e celular conforme a DRC progride^4^. Nossos achados reforçam a importância de medidas de proteção individual, como o uso de máscaras, e de estratégias coletivas, como campanhas de vacinação em massa. Doses periódicas de reforço de vacina para populações de alto risco continuam fortemente recomendadas para prevenção de doença grave, complicações de longo prazo e óbito.

Por fim, este estudo apresenta algumas limitações. Como os pacientes foram incluídos durante a fase crítica da onda da variante Ômicron na pandemia de COVID-19, entre dezembro de 2021 e maio de 2022, não foi possível controlar o momento das doses de reforço nem o tipo de vacina administrada. Diante da emergência de saúde pública, não seria ético nem factível incluir controles não vacinados ou padronizar o tipo de vacina e os intervalos de reforço. A alocação de vacinas seguiu as políticas nacionais de distribuição e dependeu da disponibilidade global de doses. Além disso, receptores de transplante sob imunossupressão crônica e pacientes em diálise representavam um grupo de risco particularmente elevado, o que impediu qualquer desvio dos esquemas vacinais recomendados. Embora o número de participantes analisados tenha sido reduzido, principalmente devido à exclusão de pacientes que desenvolveram COVID-19 durante o seguimento, essa estratégia foi necessária para evitar o efeito de confusão da infecção natural sobre as respostas imunes induzidas por vacina. Considerando esses pontos, a comparação de respostas imunes celulares entre diferentes tipos de vacina não foi viável e deve ser interpretada com cautela. Ademais, o teste de liberação de IFN-γ QuantiFERON apresentou grande número de resultados indeterminados e cinética variável durante o seguimento, particularmente entre RTR, sugerindo necessidade de interpretação cautelosa dos resultados. Esse achado provavelmente reflete tanto questões técnicas inerentes ao ensaio quanto fatores relacionados aos pacientes. O QuantiFERON requer condições de manuseio precisas, incluindo incubação imediata e centrifugação controlada, que podem afetar o desempenho do teste. Adicionalmente, a depleção de células T induzida por globulina antitimócito, a linfopenia persistente e a disfunção funcional de linfócitos T causada pela imunossupressão crônica podem contribuir para a redução da liberação de IFN-γ e, consequentemente, para resultados indeterminados^14-16^.

Estudos futuros devem explorar ensaios complementares, como ELISpot e citometria de fluxo, que podem avaliar de forma mais acurada a imunidade celular específica contra o SARS-CoV-2 em pacientes imunossuprimidos. Esses métodos quantificam respostas de linfócitos T específicos para antígenos e caracterizam subpopulações funcionais, oferecendo maior sensibilidade do que o IGRA. Entretanto, cada ensaio captura aspectos distintos da atividade de células T, e nenhum teste isolado reflete plenamente a complexidade da imunidade celular. Evidências recentes apoiam a combinação de múltiplos ensaios para aprimorar a avaliação das respostas induzidas por vacina em pacientes transplantados renais e em diálise^21^.

Do ponto de vista clínico, nossos achados apoiam a necessidade de estratégias de vacinação personalizadas para pacientes com doença renal avançada, particularmente receptores de transplante sob imunossupressão crônica. As respostas celulares limitadas e heterogêneas observadas neste estudo sugerem que esquemas vacinais padrão podem ser insuficientes para garantir proteção duradoura nessas populações. A monitorização periódica das respostas imunes humoral e celular pode auxiliar na identificação de não respondedores e orientar esquemas personalizados de reforço vacinal. Políticas nacionais de vacinação devem considerar a priorização desses grupos de alto risco para doses vacinais adicionais ou adjuvadas, bem como o acesso precoce a antivirais em casos de infecção *breakthrough*. Tais medidas podem otimizar a proteção e reduzir o risco de desfechos graves de COVID-19 em pacientes em diálise e transplantados renais.

## Conclusão

Nossos achados indicam que o uso do teste QuantiFERON para avaliar a imunidade celular induzida por vacina contra o SARS-CoV-2 apresenta limitações importantes em receptores de transplante renal e pacientes em diálise. Isso é particularmente evidente devido ao alto número de resultados indeterminados observados nesses grupos. Embora pacientes transplantados tenham recebido mais doses de reforço do que pacientes em diálise, não conseguimos identificar uma resposta imune celular clara e sustentada, o que reflete os efeitos da imunossupressão sobre a função de linfócitos T. Esses resultados ressaltam os desafios na monitorização da imunidade celular em populações imunocomprometidas e destacam a necessidade de métodos mais sensíveis e confiáveis para avaliação da imunidade protetora em grupos de alto risco, como ELISpot ou citometria de fluxo. Um esquema regular de doses de reforço vacinal é necessário para reduzir o risco de doença grave e mortalidade nessa população vulnerável.

## Data Availability

Os conjuntos de dados gerados e/ou analisados durante o presente estudo estão disponíveis mediante solicitação razoável ao autor correspondente.
